# Spontaneous intracranial hypotension due to CSF–venous fistula: Evaluation of renal accumulation of contrast following decubitus myelography and maintained decubitus CT to improve fistula localization

**DOI:** 10.1177/15910199231172627

**Published:** 2023-05-21

**Authors:** Richard I Farb, Sean T O’Reilly, Everardus J Hendriks, Philip W Peng, Eric M Massicotte, Yasmine Hoydonckx, Patrick J Nicholson

**Affiliations:** 1 Department of Diagnostic Imaging, Division of Neuroradiology, Toronto Western Hospital, University of Toronto, Toronto, Ontario, Canada; 2 Department of Anesthesiology, Toronto Western Hospital, University of Toronto, Toronto, Ontario, Canada; 3 Department of Surgery, Division of Neurosurgery, Toronto Western Hospital, University of Toronto, Toronto, Ontario, Canada

**Keywords:** Cerebrospinal fluid leak, CSF–venous fistula, digital subtraction myelography, decubitus myelography

## Abstract

**Purpose:**

Presented here is a strategy of sequential lateral decubitus digital subtraction myelography (LDDSM) followed closely by lateral decubitus CT (LDCT) to facilitate cerebrospinal fluid (CSF)–venous fistula (CVF) localization.

**Materials and Methods:**

This is a retrospective analysis of patients referred to our institution for evaluation of CSF leak. Patients with Type 1 and Type 2 leaks, and those not displaying MR brain stigmata of intracranial hypotension were excluded. All patients underwent consecutive LDDSM and LDCT. If the CVF was not localized on the first LDDSM–LDCT pair the patient returned for contralateral examinations. Images were reviewed for CVF and for accumulation of contrast within the renal pelvises expressed as a renal pelvis contrast score (RPCS) in Hounsfield units (HU).

**Results:**

Twenty-two patients were included in this study. In 21 of 22 patients (95%) a CVF was identified yielding an RPCS for the LDDSM–LDCT pair ipsilateral to the CVF ranging from 71 to 423 with an average of 146 HU. An RPCS of the negative side LDDSM–LDCT pair contralateral to a CVF was available in 8 patients and averaged 51 HU. In 4 patients the initial bilateral LDDSM–LDCT pairs did not reveal the location of the CVF however in 3 of these 4 cases the CVF was revealed on a third LDDSM repeated ipsilateral to the higher RPCS.

**Conclusion:**

The strategy of sequential LDDSM–LDCT coupled with evaluation of renal accumulation of contrast agent appears to improve the rate of CVF localization and warrants further evaluation.

## Introduction

Approximately 30% of cerebrospinal fluid leaks (CSF leaks) are due to a CSF to venous fistula (CVF)^1,2^ also known as a type 3 leak. The entity of CVF, first reported in 2014 by Schievink,^3^ represents a CSF leak in which there is an aberrant fistulous communication along a distal root sleeve between the subarachnoid space and a paravertebral vein.^2,4^ These CVFs are not associated with a spinal longitudinal extradural collection (SLEC) as the leak is beyond the spinal canal and thus rather than accumulating in the epidural space the CSF is carried away by the venous system. A CVF is commonly more challenging to localize. Currently, the best methods for localizing a CVF is with lateral decubitus digital subtraction myelography (LDDSM)^1,2,5^ or lateral decubitus dynamic CT myelography.^4,6,7^ Despite the use of these techniques a CVF may not be found in a proportion of patients suspected of harboring such a leak. The scenario of negative bilateral decubitus myelography despite clear MR imaging evidence of underlying hypotension can be a common occurrence for neuroradiologists investigating CSF leaks. In a recent series reported by Kim et al., a CVF could not be found in 38% of patients presenting with typical clinical and MR brain stigmata of a CVF, despite performance of bilateral LDDSM.^5^

We present here a modification to our imaging strategy which we have applied to patients suspected of having a CVF which in its preliminary evaluation has resulted in a higher yield for localization of a CVF.

This modified strategy consists of LDDSM with the addition of a subsequent non-dynamic CT of the spine and kidneys (delayed by approximately 20 minutes) while maintaining the same lateral decubitus CT (LDCT) positioning. This modification by virtue of the continual pooling of contrast within the lateral gutter of the thecal sac can provide information on the rate of renal excretion. This data can then be compared to a subsequent contralateral LDDSM–LDCT pair performed at a later date (which will invariably be obtained if the first LDDSM was negative) to reveal a differential rate of renal excretion of contrast. This observed differential in the rate of contrast excretion between the two decubitus positions can determine sidedness (right or left laterality) of the CVF. This helps in deciding whether to further investigate or reinvestigate a particular side of the thecal sac.

## Methods

REB approval was obtained for this retrospective evaluation. The authors did not receive support from any organization for the submitted work. The authors have no competing interests to declare that are relevant to the content of this article.

All patients who underwent DSM at our institution for evaluation of CSF hypotension from December 2016 to April 2022 were reviewed. All patients with type 1 or 2 leaks (SLEC positive patients) were excluded. Only patients who displayed typical positive MR brain findings of intracranial hypotension (Bern > 1)^8^ were included in the study population. Patients with Bern scores of 1 or less are not routinely worked up with DSM at our institution. The remaining patients who underwent LDDSM–LDCT (with a field of view including the kidneys) for attempted localization of presumed CVF were included in the study population.

## DSM and CT

LDDSM was performed in all patients similar to protocols previously described in detail with a total of 10 mL of contrast agent (Omnipaque 300; GE Healthcare, Piscataway, NJ).^2,9^ All patients were normally hydrated prior to the DSM and were NPO from midnight the night before except for medications with sips. We routinely perform DSM with local anesthesia and no intravenous sedation. No patients had recent injection of contrast within the preceding 72 hours. All DSMs were performed in a decubitus position following which patients were transferred from the angiography table to a stretcher and immediately transported to the CT suite and scanned all the while maintaining the decubitus position level to the floor with the head mildly elevated on a pillow. Following CT the patients were repositioned for comfort and transferred to the day unit for recovery and discharge. DSM was performed initially in all patients with local anesthetic and no intravenous medication or fluid. At our center we usually performed LDDSM in the right side down position first. If the first LDDSM was negative the patient returned a minimum of 3 days later for repeat LDDSM and CT on the other side. At our center we do not routinely perform a second LDDSM (on the contralateral side) if the first study is positive for CVF.

## Image review

All imaging was reviewed by two neuroradiologists reaching a consensus for all imaging criteria. All DSM images were reviewed for presence or absence of CVF. All CTs were reviewed for evidence of CVF which included possible direct visualization of the fistula or indirect evidence such as higher density in a paravertebral azygous/hemiazygos vein. CTs were also reviewed for tabulating of observed concentration of contrast agent within the renal collecting systems of both kidneys. Care was taken not confuse a renal calculus for contrast agent. A renal pelvis contrast score (RPCS) was determined as the highest Hounsfield unit (HU) obtained by applying at least three ROIs over each renal pelvis of each kidney. These measurements were performed on 3-mm thick axial sections reconstructed with soft tissue algorithm. A minimum of 0.03 cm^2^ was required for each ROI to be considered. At times the collecting systems yielded no identifiable contrast agent such that the renal pelvis could not be reliably distinguished from the adjacent unpacified renal vasculature. In these instances, a non-distinguishable pelvis was recorded as the RPCS equal to 45 HU (the average HU of vascular structures in the renal hilum). Occasionally the renal pelvis was easily distinguished as lower in density than the vascular structure of the renal hilum. The RPCS was also tabulated as being obtained in a decubitus position ipsilateral or contralateral to a known CVF when one had been found. The elapsed time from injection of contrast at DSM to the time of CT scanning was also obtained from the time stamps annotated on the images.

## Results

Review of records from our institution from April 2016 to February 2023 revealed 22 patients meeting inclusion criteria for this study in whom decubitus DSM and decubitus CT were sequentially performed and available for review. This subset of patients consisted of 8 male and 14 females with a mean age of 53 years. Data of 5 of these patients has appeared in a prior publication.^2^ The average Bern score was 6.5. In 21 of the 22 (95%) patients a CVF was eventually definitively identified.

Patients were stratified into four groups based on the number of LDDSM exams performed and availability of renal CT data.

**Figure 1.**
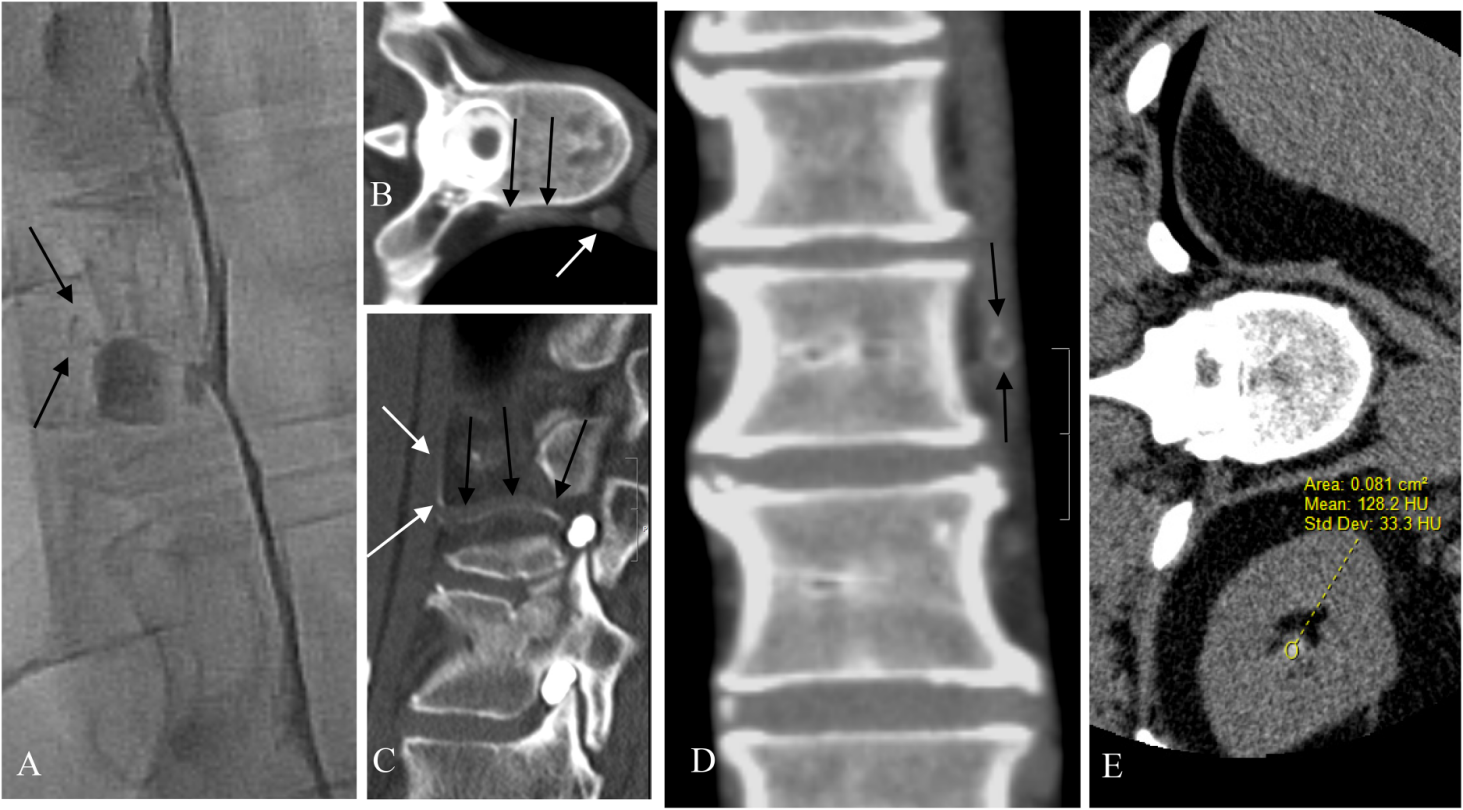
Patient 9. Showing direct CT evidence of CVF. (A) Image from left LDDSM demonstrating subtle evidence of a CVF with faint opacification of a paravertebral vein (black arrows) found retrospectively after seeing the fistula on subsequent LDCT. (B and C) Axial and sagittal reformat of the left LDCT performed immediately following the left LDDSM. Partial opacification of the paravertebral vein (black arrows) can be seen extending from the site of the fistula beside the diverticulum to the hemiazygos vein (white arrow). (D) Coronal reformat of the left LDCT showing the partial opacification of the paravertebral vein. Note the laminar flow effect with persistence of the contrast at the periphery of the vein (black arrows). (E) The left LDCT reveals indirect evidence of the presence of a CVF with early contrast agent arrival in the renal pelvis measuring 128 HU.

Group A consisted of four patients whose initial LDDSM was negative. These patients were encountered early in our experience when we did not routinely perform LDCT in DSM negative exams. Therefore, in this group of four patients RPCS data on the side contralateral to the CVF (the negative side) is not available. In these patients the second LDDSM revealed a CVF and a LDCT was also performed providing CT data for ipsilateral CVFs (Figure 1).

Group B consisted of nine patients whose initial LDDSM revealed a CVF, these patients also underwent LDCT thus providing data for LDCT ipsilateral to a CVF. As stated previously, having found a CVF on the initial decubitus study, we do not continue to examine the contralateral side at our center. Therefore, in this group of patients there is no CT data regarding the contralateral side.

Group C consisted of five patients whose initial DSM was negative for a CVF but nonetheless (as is now our current practice) also underwent LDCT of the LDDSM negative side. These patients then went on to undergo a second LDDSM and LDCT one week later on the opposite side revealing a CVF. CT data is therefore available both ipsilateral and contralateral to the CVF in these patients.

**Figure 2.**
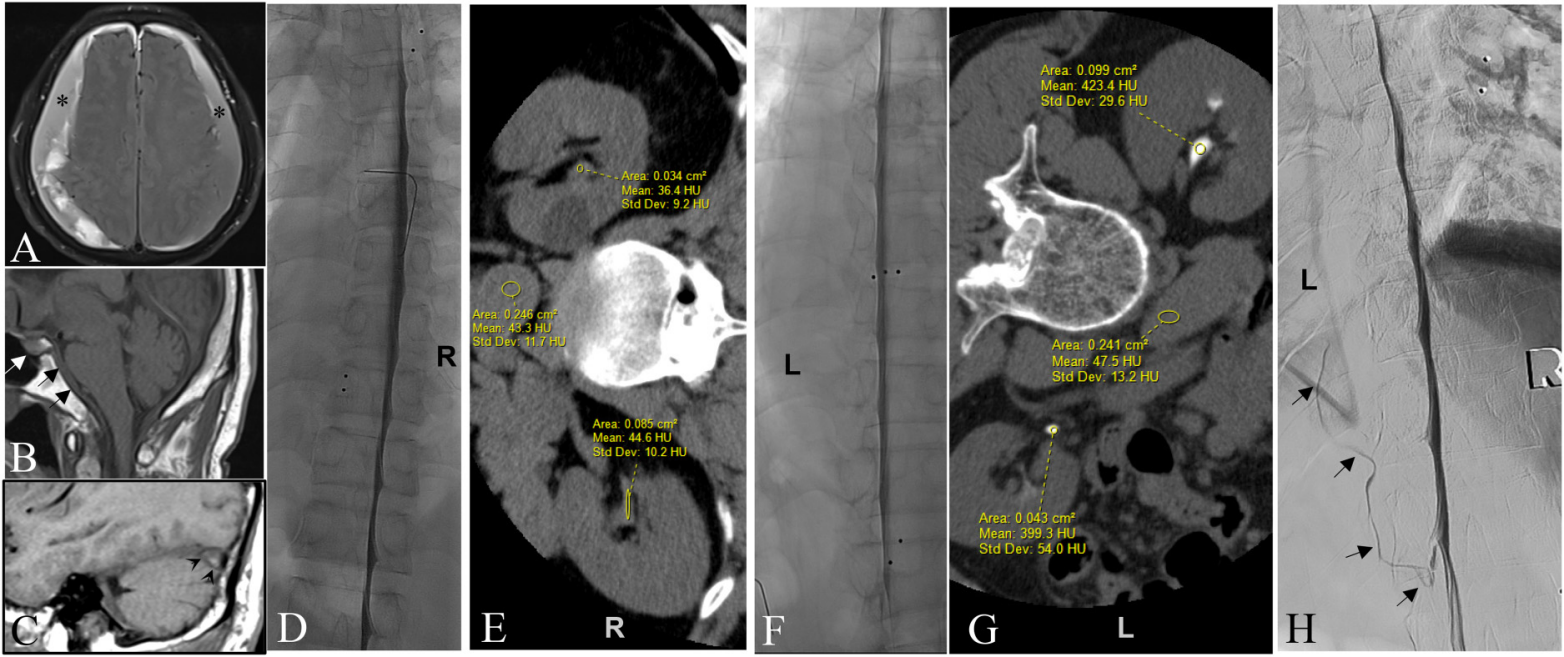
Patient 11. Showing differential rates of renal excretion associated with right versus left decubitus DSM–CT pair predicting sidedness of the culprit CVF . (A) Axial FLAIR image showing large bilateral subacute SDHs over the convexities of the brain (asterisks). (B and C) Midline and paramedian sagittal T1 images of the brain respectively showing enlargement of the pituitary (white arrow), dural thickening on the clivus (black arrows) and convex undersurface of the transverse sinus (venous distension sign) (arrowheads) as signs of intracranial hypotension despite the presence of bilateral subdural hematomas. (D) Right LDDSM showing no evidence of CVF. (E) Right decubitus CT performed immediately following the DSM showing only subtle contrast in the renal collecting systems measuring up to 45 HU . (F) Left LDDSM did not reveal a CVF. (G) Left decubitus CT performed immediately following the left LDDSM does show a high level of contrast agent within the renal collecting systems measuring up to 423 HU. The fact that contrast agent is seen in the renal pelvises immediately following DSM strongly suggest that a CVF is indeed present in this patient. Moreover, it is the disparity between these two decubitus measurements of renal contrast, i.e. much higher following the left LDDSM–LDCT pair, that further indicates that the CVF is most likely present on the left side even though it was not visualized on the original left LDDSM. This therefore compelled us to repeat left LDDSM. (H) Repeat Left LDDSM with the benefit of general anesthetic revealing the CVF arising beneath the left T10 pedicle ascending superiorly in a left paravertebral vein (black arrows).

Group D consisted of four patients who underwent complete bilateral LDDSM and LDCT which did not initially reveal a CVF but then underwent a third LDDSM repeated on the side which had previously displayed the higher RPCS (Figure 2). In three of these four patients the CVF was then found at the third LDDSM. In the fourth patient the CVF was never found despite a third LDDSM under GA and persistent elevated RPCS. This patient continues to display MR brain stigmata of hypotension with mild symptoms.

**Figure 3.**
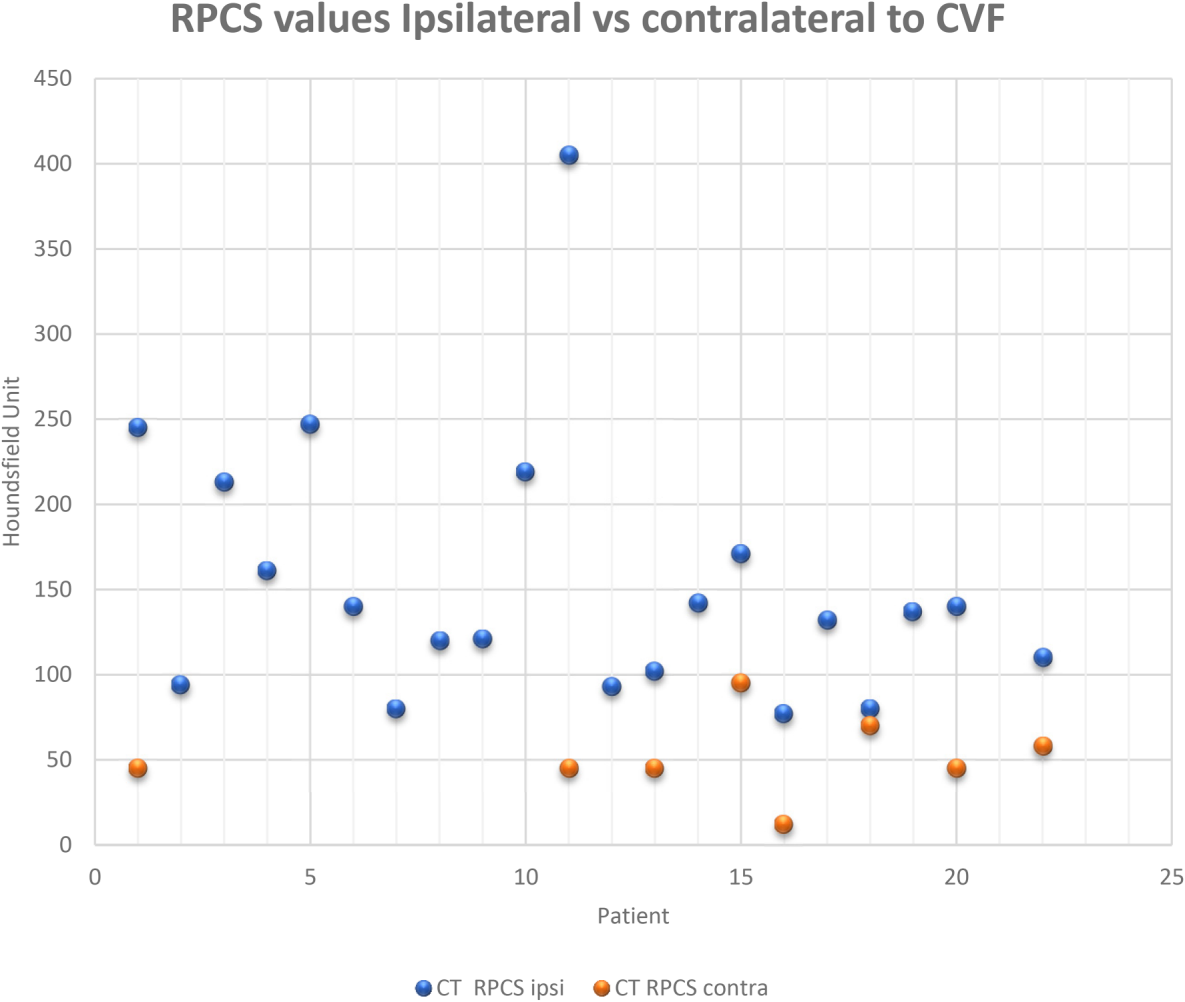
A scatter plot of each patient’s RPCS values obtained following decubitus DSM ipsilateral and contralateral (where available) relative to the side of the localized CVF. Note in the eight patients where both ipsilateral and contralateral values are obtained the ipsilateral RPCS was always higher.

In 21 patients a RPCS was obtained from the LDDSM–LDCT pair ipsilateral to the CVF. Each of these “ipsilateral” RPCSs exceeded 76 HU with a range of 77 to 423 and a mean of 151 HU. An RPCS of the negative side LDDSM–LDCT pair contralateral to a CVF, was available in 8 patients and was found to be lower than the RPCS of the LDDSM–LDCT pair ipsilateral to the fistula in all 8 patients. In each of these patients the renal accumulation of contrast was always higher when the lateral decubitus (down) positioning was ipsilateral to the fistula (Figure 3). The magnitude of the differential of the RPCS obtained following a LDDSM ipsilateral to the CVF versus the RPCS obtained following a LDDSM contralateral to the CVF (when available) varied from a 1.1- to a 9-fold increase, with a mean increase of 3.8-fold (Table 1).

**Table 1.** The 22 patients who underwent LDDSM and decubitus CT.

Group	Patient	Age	Gender	Bern score	CVF found	Location	Direct CT viz of CVF	CT RPCS ipsi	Interval DSM–CT (min)	CT RPCS contra	Interval DSM–CT (min)	Ratio RPCS i/c
A	5	59	F	4	Y	LT2	No	247	40			
	6	59	M	9	Y	RT9	No	140	36			
	7	68	F	9	Y	RT10	No	80	28			
	9	62	F	6	Y	LT9	Yes	121	27			
B	2	56	F	7	Y	LT7	No	94	35			
	3	61	M	7	Y	LT3	No	213	31			
	4	44	F	6	Y	LT9	No	161	25			
	8	61	M	5	Y	LT11	No	120	38			
	10	35	F	4	Y	RT9	No	219	15			
	12	53	M	7	Y	RT10	No	93	15			
	14	32	M	9	Y	RT10	Yes	142	22			
	17	58	F	8	Y	LT10	No	132	24			
	19	62	F	8	Y	RT1	No	137	21			
C	18	68	F	7	Y	LT7	Yes	80	30	70	18	1.1
	15	40	F	5	Y	LT3	No	171	32	95	26	1.8
	16	35	F	7	Y	LT10	Yes	77	22	12	20	5.9
	20	50	M	6	Y	LT1	No	140	29	45	22	3.1
	22	62	F	6	Y	LT8	No	110	26	58	20	1.9
D	1	47	M	6	Y	RT7	No	245	18	45	27	5.4
	11	74	M	7	Y	LT12	No	405	29	45	18	9
	13	53	F	6	Y	RT4	No	102	22	45	20	2.3
	21	31	F	4	N	n/a	No	95	18	45	22	n/a
Avg		53	14F/8M	6.6		13L/8R	4	151	25.9	51.1	21.4	3.8

*CT RPCS ipsi*  =  Renal Pelvis Contrast Score obtained on the decubitus CT ipsilateral to the side of the CVF.

*CT RPCS contra*  *=*  Renal Pelvis Contrast Score obtained on the decubitus CT contralateral to the side of the CVF.

Group A  =  Patients whose initial LDDSM was negative for CVF and didn’t undergo CT on the negative side.

Group B  =  Patients whose initial LDDSM was positive for CVF and also had decubitus CT. they did not undergo contralateral imaging.

Group C  =  Patients whose initial LDDSM was negative for CVF but also had CT on the negative side and then went on to have remaining side DDSM and CT.

Group D  =  Patients whose initial right and left LDDSM were negative for CVF and underwent a repeat LDDSM compelled by the higher CT RPCSs.j

Direct evidence of CVF, seen as contrast in paravertebral veins, was seen in only three patients when LDDSM–LDCT was done ipsilateral to the CVF (Figure 1).

The elapsed time from injection of contrast agent at DSM to CT scanning across all LDDSM–LDCT pairs ranged from 15 to 40 minutes with a mean of 25 minutes.

## Discussion

This paper builds on the work of previous authors who identified that following myelography renal collecting systems fill with contrast at a faster rate in patients with CSF leak^10–12^ compared to control patients. The results reported here not only substantiate those reports but employ a maintained decubitus position to potentiate the flow of contrast through an ipsilateral fistula into a vein accentuating the renal contrast accumulation seen at CT thus providing criteria to determine the side of the culprit CVF even though it may not have been identified at the preceding LDDSM. In the study reported here this occurred in three cases where the CVF was not seen on the initial right and left LDDSM, the LDCT renal pelvis accumulation of contrast not only clearly indicated the presence of an underlying fistula but also pointed to a suspect side based on the disparate RPCS values compelling a repeat DSM on the suspect side. This indeed resulted in identifying the CVF thus increasing the diagnostic yield of the search paradigm for localizing a CVF from 18 out of 22 (82%) to 21 of 22 (95%) in this small series. In this limited study of 22 patients the rapid accumulation of renal contrast can be used as a useful sign indicating that there is a high likelihood of an ipsilateral CVF. This in turn would then justify continued efforts with ipsilateral repeat DSM exams to localize the fistula. The results presented here suggest that LDDSM followed closely with decubitus non-dynamic CT can point to the side of the fistula. This phenomenon occurs due to the fact that the contrast is denser than CSF. Therefore, when LDDSM floods the dependent lateral gutter of the thecal sac with contrast it covers and continuously perfuses a CVF that may exist on that side enhancing the flow of contrast from the thecal sac through the fistula into the venous system which readily reaches the kidneys and concentrates in the renal collecting systems. Conversely, when the fistula is on the (high) side contralateral to the flooded gutter a diminished concentration of contrast agent is available to perfuse the fistula leading to a lower rate of concentration in the renal pelvises.

It should be noted that many factors are at play when considering the concentration of contrast in the renal pelvis after myelography. These factors include but are not limited to the initial size of the renal pelvis resulting in dilutional effects, the size of the patient, hydration state of the patient, glomerular filtration rate (GFR), time interval between injection and scanning, as well as the variability of our crude ROI measurements over the renal pelvises. Also, despite our (and the patients) best efforts there was always some variable degree of mixing of the intradural contrast in the patients while maintaining their decubitus positioning. To mitigate these potential variables, we consistently inject a total of 10 mL contrast at DSM, avoid excess hydration of patients, and attempt to perform the CTs within a consistent time window of approximately 20 minutes from time of intrathecal injection at DSM (as could be reasonably accommodated by the CT workflow). Further diminishing the effect of these variables is the fact that in several patients it was the relative difference of the RPCSs (rather than the absolute HU values) that pointed to the sidedness of the CVF.

At our center, we have routinely incorporated this principle of observing differential renal excretion based on a CVF existing ipsilateral or contralateral to the maintained decubitus position of DSM and CT. We have found this systematic methodology to be very helpful when evaluating patients strongly suspected of harboring a CVF (i.e., MR brain positive and SLEC negative patients).

A weakness of this study is the lack of a control population, that is, RPCS in subjects undergoing CT myelography for reasons other than CSF leak. However, we can draw from the experience of earlier reports which have shown a consistently very low contrast accumulation in the renal pelvises of their control groups.^10–12^ Also, the study presented here uses the internal control of the RPCS when the decubitus DSM–CT pair was performed on the side contralateral to a CVF. A potential confounding factor not accounted for in this report is the possibility of multiple or bilateral CVFs. The multiplicity of CVF has been estimated at less than 10%^13^ and is likely not a significant factor in this preliminary report but does need to be considered as this strategy is further evaluated.

In a minority of patients (3 of 22, i.e. 14%) decubitus CT following DSM revealed direct evidence of the CSF venous fistula (visualization of opacification of a paravertebral vein on CT). Furthermore, the evidence in each case was quite subtle and likely only identifiable in light of the fact that the neuroradiologist was aware of the fistula location from the DSM. This is unlike the experience reported for dynamic CT myelography^6^ and may relate to the lack of the immediate pressure elevation of dynamic injection, lack of temporal resolution with single-phase CT performed here, lower relative contrast concentrations and dissipation of the added intrathecal pressure afforded by the pre-injection “pressurization” of 10 mL of saline commonly employed by myelographers. This again speaks in favor of the theory that these fistulas are themselves dynamic and not continuously open and leaking. Understanding how they may be induced to be “active” as suggested by other authors^14^ would help in their consistent localization.

## Conclusion

In this early analysis, for patients presenting with the typical clinical and MR signs of SIH due to CVF, we have found the strategy of evaluating for renal excretion of contrast agent following sequential LDDSM–LDCT pairing to be helpful in localizing the underlying CVF. The application of this strategy has resulted in the correct prediction of laterality of the culprit CVF leading to improved diagnostic yield with eventual localization of the leak in 95% of MR positive patients in this initial small series. Further evaluation of this strategy is warranted.
